# Supplementary material for: Mapping the evolution of fertility support policies in China: A content and instrumental analysis
Source: PLoS One. 2025 Oct 9;20(10):e0332137. doi: 10.1371/journal.pone.0332137 (PMC12510515; doi:10.1371/journal.pone.0332137)
Supplement: S1 Appendix — (ZIP) [file pone.0332137.s001.zip › S1 Appendix. 226 original policy documents/13-国家人口和计划生育委员会、中国计划生育协会、中国人口福利基金会关于开展创建幸福家庭活动试点工作的指导意见(FBM-CLI-4-150860).doc]

国家人口和计划生育委员会、中国计划生育协会、中国人口福利基金会关于开展创建幸福家庭活动试点工作的指导意见

**发布部门：** [国家人口和计划生育委员会(含国家计划生育委员会)(已撤销)](https://www.pkulaw.com/law/chl?Aggs.IssueDepartment=60304) [中国计划生育协会](https://www.pkulaw.com/law/chl?Aggs.IssueDepartment=70572) [中国人口福利基金会](https://www.pkulaw.com/law/chl?Aggs.IssueDepartment=7060811) [机构沿革](javascript:void(0))

**发文字号：**人口厅〔2011〕46号

**发布日期：**2011.05.04

**实施日期：**2011.05.04

**时效性：** [现行有效](https://www.pkulaw.com/law/chl?Aggs.TimelinessDic=01)

**效力级别：** [部门工作文件](https://www.pkulaw.com/law/chl?Aggs.EffectivenessDic=XE0303)

**法规类别：** [计划生育管理](https://www.pkulaw.com/law/chl?Aggs.Category=09705)

国家人口和计划生育委员会、中国计划生育协会、中国人口福利基金会关于开展创建幸福家庭活动试点工作的指导意见
（人口厅〔2011〕46号）

各省、自治区、直辖市人口计生委、计生协、人口福利基金会，计划单列市、新疆生产建设兵团人口计生委、计生协、人口福利基金会，解放军、武警部队计划生育领导小组办公室，中直机关、中央国家机关人口计生委：
　　为深入学习实践科学发展观，贯彻落实党的十七届五中全会以及中央关于全面做好人口工作、加强和创新社会管理的一系列重要指示精神，统筹解决人口问题，促进人口长期均衡发展，国家人口和计划生育委员会、中国计划生育协会、中国人口福利基金会决定开展创建幸福家庭活动，现提出如下意见。
　　**一、**充分认识创建幸福家庭活动的重大意义
　　（一）创建幸福家庭活动是贯彻落实科学发展观、全面做好人口工作的重要内容。科学发展观的核心是以人为本。人口是影响经济社会发展的关键因素，关系改革开放和社会主义现代化建设的成功，关系中华民族的未来。我国是一个拥有13亿多人口的发展中大国，经济发展和社会管理面临的重大问题，都与人口数量、素质、结构、分布问题密切相关。要充分认识人口问题的长期性、复杂性、艰巨性，不断增强做好人口工作的自觉性和主动性。家庭是做好人口工作的重要着力点，开展创建幸福家庭活动，对于坚持计划生育基本国策，稳定低生育水平，提高人口素质，优化人口结构，引导人口合理分布，保障人口安全，促进人口与经济、社会、资源、环境协调可持续发展，具有积极而重要的作用。
　　（二）创建幸福家庭活动是构建社会主义和谐社会、提高家庭发展能力的务实举措。家庭是社会的基本细胞，家庭幸福是社会和谐的重要基础。随着人口转变和经济社会转型，我国家庭结构发生了深刻变化，家庭规模缩减，居住离散，独生子女家庭比例升高，家庭在婚姻、生育、教育、就业、医疗、养老等方面出现了许多新情况、新问题。开展创建幸福家庭活动，就是要建立健全家庭发展政策，切实促进家庭和谐幸福，加大对孤儿监护人家庭、老年人家庭、残疾人家庭、留守人口家庭、流动人口家庭、受灾家庭以及其他特殊困难家庭的扶助力度，倡导和营造家庭和美、邻里和气、社区和睦的良好氛围，为构建社会主义和谐社会奠定坚实的基础。
　　（三）创建幸福家庭活动是加强和创新社会管理、发挥群众团体和社会组织作用的有效载体。中央要求，进一步加强和完善社会管理格局，引导各类社会组织加强自身建设、增强服务社会能力，支持人民团体参与社会管理和公共服务，发挥群众参与社会管理的基础作用。开展创建幸福家庭活动，是深化人口计生综合改革的重要内容，是群众团体和社会组织围绕大局、履行责任、服务民生的具体实践。有助于创新人口和计划生育工作体制机制和手段方法，全面加强基层基础工作；有助于推动建立健全党委领导、政府负责、社会协同、公众参与的工作格局，寓管理于服务之中，不断提高人口服务和管理水平。

　　**二、**创建幸福家庭活动的指导思想、总体目标和基本原则
　　（一）指导思想。以科学发展观为统领，以综合改革为动力，坚持以人为本，充分调动政府部门、群众团体、社会组织和企业的力量，发挥人口计生工作网络健全的优势，有效整合各方面资源，推动解决城乡家庭特别是计划生育家庭在生产、生活、生育、养老等方面存在的问题，切实稳定低生育水平，统筹解决人口问题，提高家庭发展能力，促进人口长期均衡发展和社会和谐进步。
　　（二）总体目标。“十二五”期间，通过开展宣传倡导、健康促进、致富发展“三大活动”，进一步弘扬中华民族重视家庭、夫妻和睦、尊老爱幼、邻里互助等传统美德，推动男女平等、计划生育、优生优育优教等文明理念更加深入人心；提高计划生育/生殖健康水平，降低出生人口性别比，减少出生缺陷发生风险；健全有利于支持家庭发展的经济社会政策，困难家庭得到有效救助，计划生育家庭优先分享改革发展成果，家庭生活质量和幸福指数进一步提高。
　　（三）基本原则。
　　--党政领导，统筹协调。坚持把经济增长指标同人口、资源、环境、社会发展指标有机结合起来，突出家庭发展在社会主义和谐社会建设中的重要地位和作用，把创建幸福家庭活动纳入本地经济社会发展规划，纳入保障和改善民生的重要内容，确保责任到位、措施到位、投入到位、落实到位。
　　--发挥优势，服务大局。依托人口计生系统行政管理、宣传倡导、科技服务、信息综合、群众工作的公共服务网络，充分发挥其扎根基层、遍布城乡、联系群众、服务上门的特色和优势，努力提高服务家庭发展的能力，促进中央关于全面做好人口工作各项决策的贯彻落实。
　　--继承传统，开放创新。大力弘扬中华优秀传统文化中“仁、义、礼、智、信、慈、孝、和”等内容，总结推广新中国成立后特别是改革开放以来，在家庭文化建设中取得的新成果新经验，借鉴国际社会支持家庭发展的先进做法，不断丰富创建幸福家庭活动的内涵，提升活动的亲和力、影响力和生命力。
　　--试点先行，务求实效。综合考虑经济社会发展条件和工作基础、实践经验等因素，在全国先行选择一批创建幸福家庭活动试点单位，探索经验，打造品牌。试点地区要因地制宜，细化措施，真心实意为群众解难事、办实事、求实效，提高城乡家庭的参与率和满意度。

　　**三、**创建幸福家庭活动的主要内容、阶段安排和主要措施
　　（一）主要内容。创建幸福家庭活动重点围绕“文明、健康、优生、致富、奉献”五个方面展开。
　　--文明倡导。文明是幸福家庭的道德追求。引导家庭成员树立社会主义核心价值观，提高思想道德和科学文化素质，热爱祖国，关心社会，珍惜家庭，推动家庭成员之间、家庭与社会、家庭与自然的友爱、和谐。
　　--健康促进。健康是幸福家庭的基础条件。培养家庭成员科学文明、低碳环保、健康绿色的工作方式与生活习惯，提高生活保健和生殖健康的意识、能力和水平，促进家庭成员身体健康、心情愉悦，不断增强幸福感。
　　--优生优育。优生是幸福家庭的希望所在。加强人力资源开发，着力提高人口素质，开展免费孕前优生健康检查，降低出生缺陷发生风险。普及科学育儿知识，注重婴幼儿早期发展，加强青少年健康人格教育，促进人的全面发展。
　　--致富发展。致富是幸福家庭的重要保障。进一步完善各项惠民利民的经济社会政策，通过多种帮扶救助方式，支持城乡家庭特别是计划生育困难家庭发展生产、勤劳致富，实现收入增加、生活宽裕、富足祥和、安居乐业。
　　--奉献社会。奉献是幸福家庭的价值体现。强化家庭成员的公民意识和感恩情怀，增强家庭责任感和社会凝聚力，形成人人乐于扶危济困、见义勇为、养老助残、邻里互助、回报社会的良好道德风尚。
　　（二）阶段安排。创建幸福家庭活动是一项系统工程和长期任务，需要科学谋划、分步实施。“十二五”期间的工作将分三个阶段进行：
　　--启动阶段（2011年5月至12月）。主要工作：选择试点，部署任务。各试点地区宣传发动，开展活动。
　　--推进阶段（2012年1月至2015年7月）。主要工作：坚持分类指导，抓好落实。各试点地区完善政策措施，开拓创新，把活动不断引向深入。
　　--评估阶段（2015年8月至12月）。主要工作：对创建活动进行评估，总结试点经验，研究安排下一阶段任务。
　　（三）主要措施。广泛开展“三大活动”。
　　--宣传倡导活动。开展“幸福家庭中国行”，加强婚育文明和家庭文明建设，深化婚育新风进万家、关爱女孩行动、新农村新家庭计划等活动，通过广播、电视、网络、手机、报刊等大众传媒，采取文艺演出、广场活动等多种方式，广泛宣传文明进步的家庭观、幸福观。完善健康咨询电子信息服务系统，普及计划生育、生殖保健以及保护妇女、儿童、老年人、流动人口权益的法律法规知识。持续开展打击“两非”（非法进行胎儿性别鉴定和非医学需要的人工终止妊娠）行动，切实降低出生人口性别比。开展中国家庭幸福指数研究，探索建立中国家庭幸福状况评价体系，发布《中国家庭年度报告》，扩大创建幸福家庭活动的社会影响力。
　　--健康促进活动。开展生殖健康援助行动，结合创建计划生育优质服务先进单位和推进“强基提质”工程，加强人口计生队伍职业化建设，提升基层服务机构装备水平和服务能力。做好免费孕前优生健康检查工作，宣传教育和组织发动以乡村为主，检测筛查以县级为主，指导和干预以地市为主。在试点地区，积极推进人口与家庭公共服务机构建设。深入开展“生育关怀”行动。配合开展“双丝带行动”（针对农村妇女的“乳腺癌、宫颈癌”防治行动），关爱育龄妇女健康。继续开展“幸福微笑”活动，对唇腭裂儿童、烧烫伤儿童等提供免费康复帮助，提高残障儿童生活能力和生存质量。
　　--致富发展活动。深入开展“幸福工程--救助贫困母亲行动”，坚持“小额资助、直接到人、滚动运作、劳动脱贫”模式，对计划生育困难家庭提供资金帮扶，从实用技术、劳动技能、生活能力、信息获取等方面进行培训，扶助开展投资少、风险小、见效快的养殖、种植、农副产品加工等劳动致富项目。全面落实农村计划生育家庭奖励扶助制度、西部地区“少生快富”工程和计划生育家庭特别扶助制度。关注城镇困难家庭，创造条件促进就业创业。关爱流动人口家庭，促进公共服务均等化。发扬敬老、养老、助老的良好社会风尚，加强公益性养老服务设施建设，探索建立“公建民营”的养老机构，努力让计划生育家庭得实惠、有保障。
　　附件：创建幸福家庭活动试点单位名单

国家人口和计划生育委员会
中国计划生育协会
中国人口福利基金会
二○一一年五月四日

　　创建幸福家庭活动试点单位名单

　　1、北京市平谷区
　　2、天津市武清区
　　3、河北省廊坊市
　　4、山西省太原市
　　5、内蒙古自治区包头市
　　6、辽宁省大连市
　　7、吉林省长春市
　　8、黑龙江省牡丹江市
　　9、上海市闵行区
　　10、江苏省苏州市
　　11、浙江省宁波市
　　12、安徽省铜陵市
　　13、福建省漳州市
　　14、江西省新余市
　　15、山东省东营市
　　16、河南省郑州市
　　17、湖北省武汉市
　　18、湖南省长沙市
　　19、广东省广州市
　　20、广西壮族自治区百色市
　　21、海南省琼海市
　　22、重庆市渝北区
　　23、四川省德阳市
　　24、贵州省遵义市
　　25、云南省玉溪市
　　26、西藏自治区林芝地区
　　27、陕西省宝鸡市
　　28、甘肃省酒泉市
　　29、青海省格尔木市
　　30、宁夏回族自治区银川市
　　31、新疆维吾尔自治区昌吉回族自治州
　　32、新疆生产建设兵团农八师石河子市

© 北大法宝：（[www.pkulaw.com](https://www.pkulaw.com/)) 专业提供法律信息、法学知识和法律软件领域各类解决方案。北大法宝为您提供丰富的参考资料，正式引用法规条文时请与标准文本核对。
欢迎查看所有[产品和服务](https://www.pkulaw.com/helps/2.html)。法宝快讯：[如何快速找到您需要的检索结果？](https://www.pkulaw.com/helps/70.html)[法宝](https://www.pkulaw.com/helps/69.html)V6有何新特色？

扫描二维码阅读原文

**原文链接：**<https://www.pkulaw.com/chl/f062a3a1f30cae02bdfb.html>
